# Career maturity among Chinese undergraduate nursing students: associations with work values, career decision-making self-efficacy, and locus of control

**DOI:** 10.1186/s12909-026-08643-8

**Published:** 2026-01-23

**Authors:** Wenfen Zhu, Qian Wu

**Affiliations:** 1 School of Nursing, Chongqing Medical University, Chongqing, China; 2 Department of Nursing, The First Affiliated Hospital of Chongqing Medical University, Chongqing, China

**Keywords:** Work values, Career decision-making self-efficacy, Locus of control, Career maturity, Nursing college students

## Abstract

**Background:**

Career maturity is a critical developmental outcome in health professional education, reflecting students’ readiness to make informed and realistic career decisions. Despite its importance, little is known about the career maturity of Chinese nursing undergraduates, particularly regarding the roles of work values, career decision-making self-efficacy (CDMSE), and locus of control. Guided by Social Cognitive Career Theory, this study examined the associations among these factors.

**Methods:**

A multicenter cross-sectional survey was conducted from May to July 2023 in five Chinese medical universities. A total of 726 undergraduates completed validated instruments: the Career Maturity Scale, Work Values Scale, College Student CDMSE Scale, and Internality, Powerful Others, and Chance Scale. Descriptive statistics, Pearson’s correlations, multiple linear regression, and structural equation modeling were used for analysis.

**Results:**

Participants demonstrated a moderate level of career maturity (M = 3.18,SD = 0.38).Correlation analyses showed that career maturity was positively associated with work values, internal locus of control, and career decision-making self-efficacy (CDMSE), and negatively associated with chance.Structural equation modeling indicated that CDMSE showed the strongest positive association with career maturity, followed by work values and internal locus of control, whereas chance was negatively associated. The final model explained approximately 33% of the variance in career maturity.

**Conclusions:**

Chinese nursing undergraduates demonstrated moderate career maturity shaped by psychological resources and personal beliefs. Targeted interventions that enhance work values, strengthen decision-making self-efficacy, and promote internal control may support students’ career readiness and sustained professional engagement. While focused on nursing, these findings also offer insights for health professional education more broadly, with implications for designing educational strategies that foster workforce sustainability.

## Introduction

Career maturity is a key developmental construct in nursing education, reflecting students’ readiness to make informed and realistic career choices [1, 2]. It has been shown to predict long-term job satisfaction, career stability, and social adjustment [3, 4]. Among nursing students, higher career maturity may also increase their motivation to remain in the profession [5]. Currently, a shortage and high turnover rate among nurses are widespread problems in many countries [6, 7]. The World Health Organization predicts a shortage of 10 million healthcare workers by 2030 [8]. Career education can enhance their career maturity [1].Therefore, enhancing the career maturity of future nurses has become an educational imperative, and nursing education must begin at the undergraduate level by focusing on the development of students’ career decision-making abilities and professional identity.These challenges are not unique to nursing; developing career maturity is a shared imperative across health professional education to ensure a sustainable and competent workforce.

Career maturity reflects an individual’s readiness for career decision-making, aligning with their career development. It encompasses attitudes, competencies, and knowledge related to career planning, including goal setting, decision-making, and occupational exploration [1, 9]. Prior research has identified influencing factors such as gender, socioeconomic status, race, and locus of control [10–12]. For example, the career maturity level of middle school students substantially varies with family income [13]. High school students with an internal locus of control positively perceive their own actions, contributing to their success at work [12]. A study conducted in Korea on the career maturity of social work students observed that intrinsic work values negatively affect purpose maturity but positively affect confidence maturity.By contrast, external work values positively affect both purpose and confidence maturity [14]. However, the career maturity of nursing college students remains largely unexplored.

This study is also informed by Social Cognitive Career Theory (SCCT), which emphasizes the joint role of self-efficacy beliefs, outcome expectations, and contextual factors in shaping career-related behaviors and adaptive outcomes [15, 16].Within this framework, career decision-making self-efficacy (CDMSE) reflects self-efficacy beliefs, work values represent outcome expectations and value appraisals, and locus of control captures individual beliefs about agency and contextual influences. Career maturity can therefore be conceptualized as an adaptive outcome of these interacting processes, making SCCT a suitable framework for interpreting the relationships examined in this study.Accordingly, CDMSE corresponds to the self-efficacy component of SCCT, work values reflect students’ outcome expectations regarding the meaning and rewards of nursing work, and locus of control represents perceived personal agency and sensitivity to contextual influences. From an SCCT perspective, career maturity may be viewed as an adaptive career outcome emerging from the interplay of these cognitive and contextual factors.

Work values refer to individuals’ beliefs and priorities related to work, including intrinsic factors (e.g., personal hgrowth opportunities and recognition) and extrinsic factors (e.g., salary, job security and prestige) [17–19]. These values shape students’ expectations about nursing as a career and influence their commitment to the profession.Although prior studies suggest a correlation between work values and career maturity [20, 21], few have examined this relationship specifically in nursing education settings.

Career decision-making self-efficacy (CDMSE) denotes an individual’s confidence in their ability to make career decisions, including areas such as gathering occupational information, self-appraisal, planning, selecting goals, and problem-solving [22]. CDMSE is a vital element in career choice and development [23]. Previous studies have indicated a close relationship between CDMSE and career maturity in college students [24, 25]. Similarly, career maturity is associated with CDMSE among higher education students [26]. However, the specific nature of the relationship between CDMSE and career maturity levels in nursing students is not well-established.

The locus of control concept, based on social learning theory and the psychological theory of career development, explains an individual’s perceived impact on achieving reinforcement [10, 27] It describes whether individuals attribute outcomes to factors within their control (internal locus of control) or to external circumstances beyond their control [28]. Levenson expanded this concept to include three dimensions: personal internality actions, powerful others, and chance (IPC), distinguishing between internal and external loci of control [29]. The locus of control is linked to an individual’s career maturity, with research indicating that college students with an internal locus of control tend to have higher levels of career maturity than those with an external locus of control [9, 27, 30].This finding suggests that locus of control could influence career maturity among nursing students.

Taken together, these factors(work values, CDMSE, and locus of control)may collectively shape the career maturity of undergraduate nursing students, but their combined influence has not been systematically examined in the Chinese context.Therefore, this study aimed to assess the level of career maturity among Chinese undergraduate nursing students and explore its associations with work values, career decision-making self-efficacy, and locus of control.By identifying these influencing factors, the study seeks to inform the development of targeted educational strategies to support students’ career readiness and professional development.

## Methods

### Design and participants

In this cross-sectional study, we used convenience sampling to recruit 797 undergraduate nursing students from five medical schools located in Shanxi, Liaoning, Shandong and Chongqing in China. All participants were enrolled in full-time undergraduate nursing programs for more than six months at the time of the study. Students not enrolled in full-time studies were excluded. This study was approved by the Ethics Committees of the participating institutions, and all procedures complied with the ethical standards of human research.

### Data collection

Data were collected from May to July in 2023.An electronic questionnaire was administered using a web-based platform. Five nursing faculty members from the respective medical schools were trained to serve as investigators and distribute the questionnaires through the WeChat platform. Before the survey commenced, the investigators informed the nursing students regarding the survey’s purpose and inclusion criteria. Students were provided the option to participate or withdraw from the survey at any time without any repercussions. All eligible and willing nursing students provided informed consent before completing the questionnaire anonymously. The electronic questionnaire required approximately 10 min for completion. Afterward, the researchers were responsible for examining and discarding invalid questionnaires on the basis of the following criteria: (1) submissions completed in less than 5 min or more than 20 min; (2) uniform answers or a regular pattern in responses across all items (e.g., repeated sequences such as 1, 2, and 3); and (3) missing items in the questionnaire.

### Instruments

Data were collected using a self-reported questionnaire that included sections for sociodemographic information, the Career Maturity Scale (CMS), Work Values Scale (WVS), College Student CDMSE Scale, and The Internality, Powerful Others, and Chance Scale (IPC).

### Sociodemographic information

This section included age, sex, academic year, place of residence (urban/rural), and whether the student was an only child.

### The career maturity scale (CMS)

CMS was initially proposed by Lee [31] based on Crites’ concepts [32]. Zhang et al. [33]adapted and translated it into Chinese to suit the characteristics of Chinese university students. The Chinese version of the CMS consists of 34 items across six subscales: goal orientation (*n* = 8), confidence (*n* = 6), values (*n* = 6), career independence (*n* = 4), rational dependence (*n* = 4), and reference (*n* = 6). Each item is rated on a 5-point Likert scale ranging from 1 (strongly disagree) to 5 (strongly agree), with negatively phrased items scored in reverse. The total scores range from 34 to 170, where higher scores indicating greater career maturity. The CMS total score was computed by summing the scores of all 34 items. Subscale scores were calculated by summing the scores of the items corresponding to each subscale.The Chinese CMS was reported to have strong reliability and validity [33].In the present study, the Cronbach’s α for the total scale was 0.78, and the α coefficients for the six subscales were 0.70 ( goal orientation), 0.71 (confidence), 0.70 (values), 0.72 (career independence), 0.70 (reliance on family and friends), and 0.70 (reference).The subscales measure various aspects of career maturity: goal orientation assesses clarity regarding future career goals; confidence evaluates an individual’s belief in their career success; values pertain to career-related beliefs and priorities, such as social status and financial rewards; career independence gauges an individual’s autonomy in career decision-making; reliance on family and friends measures reliance on friends or family’s opinions in decision-making; and reference assesses the extent to which individuals model their career decisions on others.

### Work values scale (WVS)

Nursing students’ work values were assessed using the WVS developed by Ling et al. [34]. Ling et al. [34] conducted a systematic analysis of work values, drawing on Herzberg’s two-factor theory of motivation. They factor-analyzed value entries from the literature and created the WVS, which includes 22 items across three dimensions: prestige status (*n* = 9), health care factors (*n* = 6), and self-development factors (*n* = 7). Responses to the items were scored on a 5-point Likert scale ranging from 1 (not important) to 5 (very important). The total WVS score was computed by summing the scores of all 22 items. Subscale scores were calculated by summing the scores of the items corresponding to each dimension. Higher subscale scores indicate that the corresponding work values are more significant in the respondents’ career choices. The Chinese version of the WVS was found to be highly reliable and valid for use with college students [34].In this study, the Cronbach’s α for the total scale was 0.90, and the α coefficients for the three subscales were 0.87 (prestige status), 0.85 (health care factors), and 0.81 (self-development factors).

### College student career decision-making self-efficacy (CDMSE) scale

Nursing students’ CDMSE was measured using the College Student CDMSE scale developed by Peng et al. based on the foundational CDMSE scale by Betz and Taylor [35, 36]. This scale, known for its high reliability and validity, comprises 39 items across five dimensions: self-appraisal (*n* = 6 items), goal selection (*n* = 9), occupational information (*n* = 9), planning (*n* = 8), and problem-solving (*n* = 7). Responses were rated on a 5-point Likert scale ranging from 1 (not confident at all) to 5 (completely confident). The total scores range from 39 to 195, with higher scores indicating better CDMSE. The total score was computed by summing the scores of all 39 items, and subscale scores were calculated by summing the items corresponding to each dimension.For this study, the scale’s Cronbach’s alpha was 0.97. The α coefficients for the five subscales were 0.84 (self-appraisal), 0.89 (occupational information), 0.87 (goal selection), 0.86 (planning), and 0.84 (problem-solving), indicating excellent internal consistency across all dimensions.

### The internality, powerful others, and chance (IPC)scale

The Chinese version of the IPC scale, developed by Levenson [29] and revised by Wang et al. [37], was used to assess the locus of control among nursing students. The IPC scale comprises 24 items across three dimensions: internality (*n* = 8), powerful others (*n* = 8), and chance (*n* = 8). Items were rated on a 6-point Likert scale ranging from − 3 (strongly disagree) to 3 (strongly agree). For each dimension, item scores were summed (raw range: −24 to 24) and then 24 points were added to offset negative values, yielding a final subscale score ranging from 0 to 48.Accordingly, each subscale score ranges from 0 to 48, with higher scores indicating stronger beliefs consistent with the respective dimension.The scale has demonstrated good reliability and validity in Chinese samples [37].In this study, the Cronbach’s α for the total scale was 0.85, and the α coefficients for the internality, powerful others, and chance subscales were 0.70, 0.75, and 0.72, respectively.The internality dimension measures the extent to which individuals believe that their life or studies are under their own control. Powerful others assesses the degree to which individuals perceive their life or studies to be controlled by others. Chance evaluates the extent to which individuals attribute their life or learning outcomes to luck or fate.

To enhance consistency and avoid mixed score formats, summed total scores were used for all observed-variable analyses (descriptive statistics, correlations, and regression). For SEM, latent constructs (CMS, WVS, and CDMSE) were indicated by their subscale scores, whereas the three IPC dimensions were modeled as observed predictors. Mean scores are reported only for descriptive comparability across measures with different score ranges; for all metrics, higher scores indicate higher levels of the corresponding constructs.

### Data analysis

All data analyses were conducted using SPSS 26.0 and AMOS 24.0. Descriptive statistics were applied to analyze the levels of career maturity, work values, CDMSE, and locus of control. To identify differences in career maturity across various sociodemographic factors, one-way ANOVA or independent sample t tests were employed. Pearson correlation coefficients were used to explore relationships among the main variables.To assess potential common method bias, we conducted Harman’s single-factor test, entering all items from the CMS, WVS, CDMSE, and IPC scales into an unrotated exploratory factor analysis.

Multiple linear regression, with career maturity as the dependent variable, was used to examine the independent effects of work values, career decision-making self-efficacy (CDMSE), and locus of control after controlling for sociodemographic factors.This analysis also provided foundational evidence for subsequent structural equation modeling. Model 1 incorporates sociodemographic variables and work values; Model 2 adds career decision-making self-efficacy; Model 3 finally incorporates the locus of control variable.Subsequently, a two-step Structural Equation Modeling (SEM) approach was employed. First, Confirmatory Factor Analysis (CFA) was conducted to evaluate the convergent and discriminant validity of the measurement model. Latent constructs were specified using subscale indicators, and the three IPC dimensions (internality, powerful others, and chance) were entered as observed predictors of career maturity. Convergent validity was judged based on standardized factor loadings, composite reliability (CR), and average variance extracted (AVE), while discriminant validity was tested using the Fornell–Larcker criterion. Next, the structural model was specified to examine the associations between career maturity and work values, career decision-making self-efficacy (CDMSE), and locus of control. A two-tailed p-value of less than 0.05 was considered statistically significant.

## Results

### Sample features

We collected 797 questionnaires. Of these, 19 were submitted within 5 min, 10 required longer than 20 min, 15 had identical scores across all items, 13 contained incomplete information, and 14 featured regular patterns in responses (e.g., repeated sequences of 1, 2, and 3). After excluding these, 726 questionnaires were deemed valid for analysis, resulting in an effective response rate of 91.1% (726/797). Among the 726 participants, 93.5% were female and 6.5% were male. Regarding academic year, 151 (20.8%) were in Year 1, 217 (29.9%) in Year 2, 254 (35.0%) in Year 3, and 104 (14.3%) in Year 4(Table 1). Levene’s test indicated that the assumption of homogeneity of variances was violated for academic year (*p* < .05).Therefore, Welch’s ANOVA was used. The results showed significant differences in career maturity among the four academic years, Welch’s F(3,323.71) = 9.13, *p* < .001, with higher-grade students reporting higher levels of career maturity(Table 1).

**Table 1:** Characteristics and differences in participants’career maturity[M(SD)] (*n* = 726)

Variables	*N*(%)	Total career maturity	t/F	*P*
Gender				
Female	679(93.5)	107.94 ± 9.44	t = 0.962	0.336
Male	47(6.5)	109.32 ± 10.44		
Academic years				
Year 1	151(20.8)	105.28 ± 8.05	Welch F(3, 323.71) = 9.13	< 0.001
Year 2	217(29.9)	107.35 ± 9.30		
Year 3	254(35.0)	109.19 ± 9.42		
Year 4	104(14.3)	110.59 ± 10.96		
Only-child or no				
Only-child	232(32.0)	108.18 ± 10.07	t = 0.295	0.768
No	494(68.0)	107.96 ± 9.24		
District of residence				
Urban	431(59.4)	108.26 ± 9.55	t = 0.791	0.425
Rural	295(40.6)	107.69 ± 9.45		

Levene’s test indicated unequal variances for academic year (*p* < .05); therefore, Welch’s ANOVA was applied

### The level of career maturity, work values, CDMSE, and locus of control

Table 2 presents descriptive statistics for career maturity, work values, career decision-making self-efficacy (CDMSE), and locus of control. Overall, nursing students demonstrated a moderate level of career maturity (M = 3.18, SD = 0.38), with career independence highest (M = 3.48, SD = 0.54) and confidence lowest (M = 2.83, SD = 0.56). Work values were relatively high (M = 3.59, SD = 0.54) and CDMSE was moderate (M = 3.03, SD = 0.65), while locus of control indicated higher internality (M = 3.61, SD = 0.71) than chance (M = 2.82, SD = 0.78).

**Table 2:** Scores for career maturity, work values, career decision-making self-efficacy and locus of control

Variables	Items	Score range	Total score	Mean score
Total career maturity	34	34–170	108.03 ± 10.29	3.18 ± 0.38
Goal-Orientation	8	8–40	25.48 ± 3.36	3.19 ± 0.42
Confidence	6	6–30	16.99 ± 3.39	2.83 ± 0.56
Value	6	6–30	19.04 ± 3.20	3.17 ± 0.53
Career independence	4	4–20	13.90 ± 2.15	3.475 ± 0.54
Reliance on family and friends	4	4–20	12.84 ± 2.38	3.21 ± 0.60
Reference	6	6–30	19.78 ± 2.35	3.30 ± 0.39
Total work values	22	22–110	78.90 ± 11.92	3.59 ± 0.54
Prestige status	9	9–45	28.29 ± 6.56	3.14 ± 0.73
Health care	6	6–30	23.30 ± 3.79	3.88 ± 0.63
Self-development	7	7–35	27.31 ± 4.63	3.90 ± 0.66
Total career decision-making self-efficacy	39	39–195	118.07 ± 25.41	3.03 ± 0.65
Self-appraisal	6	6–30	18.63 ± 4.33	3.11 ± 0.72
Occupational information	9	9–45	27.91 ± 6.32	3.10 ± 0.70
Goal selection	9	9–45	27.06 ± 6.08	3.01 ± 0.68
Planning	8	8–40	23.63 ± 5.51	2.95 ± 0.69
Problem-solving	7	7–35	20.86 ± 4.75	2.98 ± 0.68
Total locus of control	24	24–136	74.27 ± 14.86	3.09 ± 0.62
Internality	8	0–48	28.90 ± 5.67	3.61 ± 0.71
Powerful others	8	0–48	22.86 ± 6.48	2.86 ± 0.81
Chance	8	8–40	22.52 ± 6.23	2.82 ± 0.78

### Correlation analysis

Harman’s single-factor test indicated that the first unrotated factor explained 17.56% of the total variance, which is below the suggested threshold of 40%, suggesting that common method bias was not a serious concern in this study.As shown in Table 3, total career maturity was positively correlated with total work values (*r* = .302,*p* < .01), total career decision-making self-efficacy (CDMSE; *r* = .390, *p* < .01), and internality (*r* = .225, *p* < .01), and negatively correlated with powerful others (*r*= –.146, *p* < .01) and chance (*r*=–.160, *p* < .01).

**Table 3:** Correlations between career maturity, work values, career decision-making self-efficacy and locus of control (*N* = 726)

Variables	Total career maturity	Total work values	Total career decision-making self-efficacy	Internality	Powerful others	Chance
Total career maturity	1					
Total work values	0.302**	1				
Total career decision-making self-efficacy	0.390**	0.283**	1			
Internality	0.225**	0.213**	0.360**	1		
Powerful others	−0.146**	0.080*	0.023	0.339**	1	
Chance	−0.160**	0.042	0.038	0.383**	0.694**	1

* *p* < .05,,** *p* < .01

### Multivariate regression analysis

Multiple linear regression analysis was conducted to identify predictors of career maturity. In Model 1, gender, academic year, only-child status, residence, and total work values were entered.Model 2 added total CDMSE, both work values and CDMSE were significantly predicted career maturity (β = 0.202 and β = 0.314, respectively; both *p* < .001), and R² increased from 0.118 to 0.204 (ΔR² =0.087). In Model 3, when the three locus-of-control dimensions were entered, internality remained a positive predictor (β = 0.187, *p* < .001), whereas powerful others (β=–0.098, *p* = .031) and chance (β=–0.175, *p* < .001) were negative predictors; work values (β = 0.194, *p* < .001) and CDMSE (β = 0.261, *p* < .001) also remained significant. The final model explained 26.1% of the variance in career maturity (R² =0.261).Table 4 displays the regression results.To assess potential multicollinearity, variance inflation factors (VIFs) were calculated for all regression models. All VIF values ranged from 1.01 to 2.05, indicating that multicollinearity was not a concern.

**Table 4:** Multiple linear regression analyses for factors of career maturity

	Model 1					Model 2					Model 3				
Variables	β	t	P	VIF	95% CI	β	t	P	VIF	95% CI	β	t	P	VIF	95% CI
Gender	− 0.022	− 0.620	0.536	1.010	[− 3.509, 1.825]	0.007	0.203	0.839	1.019	[− 2.282, 2.808]	− 0.007	-0.228	0.820	1.027	[− 2.753, 2.181]
Academic year	0.157	4.461	< 0.001	1.013	[0.859, 2.210]	0.109	3.202	0.001	1.040	[0.410, 1.710]	0.085	2.572	0.010	1.064	[0.197, 1.466]
Only-child or no	− 0.020	− 0.543	0.587	1.094	[− 1.870, 1.060]	− 0.017	− 0.484	0.629	1.094	[− 1.734, 1.048]	− 0.017	-0.493	0.622	1.096	[− 1.683, 1.007]
District of residence	− 0.004	− 0.104	0.917	1.096	[− 1.466, 1.318]	− 0.022	− 0.624	0.533	1.100	[− 1.745, 0.904]	− 0.025	− 0.744	0.457	1.100	[− 1.764, 0.795]
Total work values	0.288	8.151	< 0.001	1.015	[0.174, 0.284]	0.202	5.788	< 0.001	1.099	[0.106, 0.216]	0.194	5.695	< 0.001	1.123	[0.101, 0.208]
Total career decision-making self-efficacy	-	-	-	-	-	0.314	8.881	< 0.001	1.132	[0.092, 0.144]	0.261	7.198	< 0.001	1.273	[0.071, 0.124]
Internality	-	-	-	-	-	-	-	-	-	-	0.187	4.914	< 0.001	1.402	[0.188, 0.438]
Powerful others	-	-		-	-	-	-	-	-	-	− 0.098	-2.157	0.031	2.000	[− 0.275, − 0.013]
Chance	-	-	-	-	-	-	-	-	-	-	− 0.175	-3.801	< 0.001	2.045	[− 0.404, − 0.129]
R	0.343					0.452					0.511				
R^2^	0.118					0.204					0.261				
ΔR²	--					0.087					0.057				
F	19.083					30.768					28.165				

β = Standardized coefficients

### Structural equation modeling (SEM)

A two-step structural equation modeling (SEM) approach was employed, beginning with evaluation of the measurement model followed by testing of the structural model. Confirmatory factor analysis (CFA) was conducted to assess the adequacy of the latent constructs, including work values, career decision-making self-efficacy (CDMSE), and career maturity. Model fit was evaluated using multiple indices, including the Goodness-of-Fit Index (GFI), Adjusted Goodness-of-Fit Index (AGFI), Incremental Fit Index (IFI), Tucker–Lewis Index (TLI), Comparative Fit Index (CFI), and the Root Mean Square Error of Approximation (RMSEA).The measurement model demonstrated acceptable fit to the data (χ²/df = 5.44, GFI = 0.93, AGFI = 0.90, IFI = 0.95, TLI = 0.94, CFI = 0.95,RMSEA = 0.078). Standardized factor loadings ranged from 0.59 to 0.72 for work values and from 0.91 to 0.94 for CDMSE. For career maturity, standardized loadings for the five retained subdimensions ranged from 0.41 to 0.70, whereas the “Reference” subdimension showed a markedly low loading (0.30). During CFA, the “Reference” subdimension was therefore excluded from the measurement model (despite acceptable internal consistency; Cronbach’s α = 0.70). Consequently, career maturity in the SEM was indicated by the remaining five subdimensions: goal orientation, confidence, values, career independence, and relational dependence.Convergent validity was assessed using standardized factor loadings, composite reliability (CR), and average variance extracted (AVE). CR values exceeded 0.70 for work values and CDMSE, while career maturity demonstrated relatively lower but acceptable CR and AVE values(CR = 0.61; AVE = 0.24), consistent with its multidimensional nature. Discriminant validity was evaluated using the Fornell–Larcker criterion and was supported, as the square roots of the AVE for all latent constructs exceeded the corresponding inter-construct correlations (|r|≤0.48). Together, these findings indicate acceptable convergent and discriminant validity of the measurement model.

**Fig. 1:**
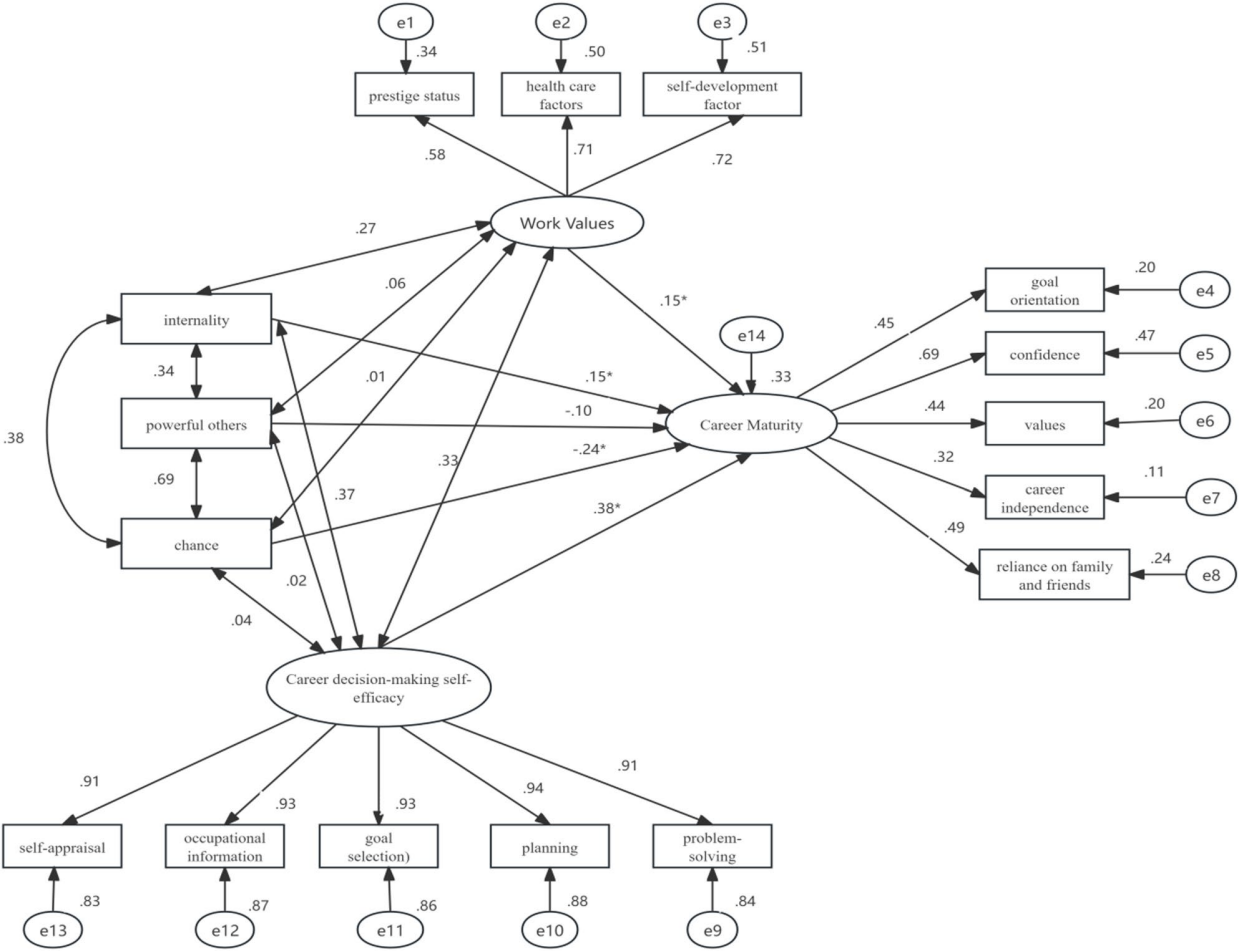
Structural equation modeling. Note: Goodness-of-fit indices:χ²/df = 4.57, GFI =.93, AGFI=.90, IFI=.95, TLI=.93, CFI=.95, RMSEA=.070;*All path coefficients are standardized estimates,p < 0.01

The structural equation model demonstrated an acceptable overall fit to the data (χ²/df = 4.57, GFI = 0.93, AGFI = 0.90, IFI = 0.95, TLI = 0.93, CFI = 0.95, RMSEA = 0.070). All factor loadings were statistically significant and in the expected directions, supporting the adequacy of the measurement model.As shown in Fig. 1, career decision-making self-efficacy (CDMSE) exhibited the strongest positive association with career maturity (β = 0.38, *p* < .001), followed by work values (β = 0.15, *p* = .006). Regarding locus of control, internality was positively associated with career maturity (β = 0.15, *p* = .004), whereas chance showed a significant negative association (β=−0.24, *p* < .001). The path from powerful others to career maturity was negative but did not reach statistical significance (β=−0.10, *p* = .091).Collectively, work values, career decision-making self-efficacy, and locus of control accounted for approximately 33% of the variance in career maturity (R²=0.33). These results indicate that nursing students’ career maturity is jointly associated with their efficacy beliefs, work-related value orientations, and perceived control beliefs, with career decision-making self-efficacy emerging as the most influential factor.Overall, the SEM findings extend the results of the correlation and regression analyses by simultaneously modeling latent constructs and accounting for measurement error, thereby providing a more robust test of the hypothesized relationships.

## Discussion

According to the average career maturity score in this study, nursing students’ career maturity was at a medium level, aligning with the findings of Cheng et al. and Lyu et al. [38, 39]. In this study, the students scored highest in “career independence”, whereas they scored lowest in “confidence”and “values”. These results suggest that although nursing students demonstrate autonomy in career decision-making, their confidence and value clarity remain relatively underdeveloped. This pattern may reflect a transitional stage of career development, in which students possess basic decision-making independence but have not yet fully internalized stable career values or confidence.

In China, employment competition among university graduates is intense [40], while the nursing profession maintains relatively stable workforce demand due to population aging and growing healthcare needs [41].Within this context, students’ early career choices may be shaped not only by personal interests but also by practical considerations and broader social influences.Previous studies have indicated that some nursing students choose the profession perceiving it as an “easy job market entry,” but they lack a thorough understanding of the nursing field [42]. Clinical challenges and unfavorable societal perceptions of nursing (e.g., heavy workload, low status, limited remuneration) may jointly undermine students’ confidence in this career choice [42]. This situation may indirectly contribute to the high turnover rate among nurses. Nursing students’ career maturity is associated with their career satisfaction [43]and the problem of nurse shortages [44], both of which substantially affect the quality of care provided by nurses [45]. Thus, it is crucial to devise interventions and strategies aimed at enhancing the career maturity levels of nursing students, which may improve their career satisfaction and address the problem of nurse shortage.

Work values showed a consistent positive association with career maturity across the regression and SEM results, indicating that higher work values are associated with increased career maturity in nursing students.Within Social Cognitive Career Theory (SCCT), work values can be conceptualized as outcome expectations and value appraisals that shape motivation for career exploration and goal setting [15, 16]. When students believe that nursing can meet valued goals such as achievement, security, and social contribution, they may be more willing to invest effort in exploring career options, clarifying goals, and engaging in planning behaviors, thereby supporting higher career maturity. This finding aligns with those of previous studies conducted in university students [20] that have established college students’ work values as predictors of their career maturity [21]. In addition, work values affect nursing students’ employment behavior and nurses’ work engagement [46–48], with both employment behavior [5] and work engagement [49]being linked to career maturity. A higher level of career maturity in college students enhances their employment readiness and competitiveness in the job market [50].Therefore, these findings suggest that educators should help students clarify and internalize work-related values that align with nursing practice, so that value appraisal can translate into sustained career planning and commitment.

Career decision-making self-efficacy (CDMSE) in this study was at a moderate level, consistent with previous findings [51]. CDMSE emerged as the strongest correlate of career maturity, particularly in the SEM, where it showed the largest standardized association with career maturity. This pattern accords with Social Cognitive Career Theory (SCCT), which posits self-efficacy as a central driver of adaptive career behaviors and outcomes [15, 16]. Students with greater confidence in self-appraisal, information seeking, goal selection, planning, and problem-solving are more likely to translate intentions into concrete decision-making actions and to persist when facing uncertainty or barriers. Previous studies similarly identify CDMSE as a key factor associated with nursing students’ career development and maturity [52]. Thus, beyond promoting positive values or favorable control beliefs, strengthening students’perceived competence in career decision processes appears particularly critical for improving career maturity. Therefore, educational efforts can target key CDMSE facets (e.g., self-appraisal, information seeking, goal setting, planning, and problem-solving) to support career maturity.

Regarding locus of control, internality was positively associated with career maturity, whereas chance showed a negative association; the direct path from powerful others to career maturity was negative but did not reach statistical significance in the SEM.In the context of Chinese nursing education, this pattern may reflect students’ heightened sensitivity to external evaluations, institutional expectations, and family influences, which could undermine their sense of agency and limit proactive career exploration.These findings suggest that a stronger internal control orientation is associated with higher career maturity. By contrast, chance represent external control factors [29], with externally controlled individuals often characterized by a sense of powerlessness, limited self-awareness, and a decreased drive for achievement [53], which may hinder career maturity [54, 55].Together, these results indicate that internality and chance are directly associated with career maturity. In addition, consistent with SCCT, it is plausible that locus of control may be linked to career maturity partly through students’CDMSE. However, this potential pathway was not explicitly tested in the current cross-sectional model and therefore warrants further examination in future research.Within the SCCT framework, locus of control reflects individuals’ perceived agency and sensitivity to contextual influences, which may shape how confidently students engage in career decision-making and planning processes. From an educational perspective, these findings suggest that strengthening students’ sense of agency and internal control may support their career maturity development. Nursing faculty should aim to empower students by giving them more control, authority, and competence [56].Within the nursing curriculum, students should be encouraged to set their own goals, pursue interests, and engage collaboratively with peers and faculty, which can collectively nurture their sense of internal control [57].Similar strategies could be incorporated into medical, pharmacy, or allied health education to enhance students’ agency and professional autonomy.

These findings can be interpreted within the framework of Social Cognitive Career Theory (SCCT), which highlights the interplay of self-efficacy, outcome expectations, and contextual influences in shaping adaptive career outcomes [15, 16]. In our study, CDMSE reflects self-efficacy, work values represent outcome expectations, and locus of control captures beliefs about agency and external influences. The positive roles of self-appraisal, occupational information, and goal selection, together with the benefits of an internal locus of control, align closely with SCCT. Importantly, the SEM results indicate that work values, locus of control, and CDMSE are jointly related to career maturity, with CDMSE demonstrating the strongest association.Thus, SCCT provides a coherent explanation for how psychological resources and contextual beliefs jointly shape nursing students’ career maturity and offers a useful basis for educational interventions.By situating nursing education findings within SCCT, this study also contributes to a broader understanding of career development across diverse health professional education settings.

Demographic variables such as gender, region of residence, and being an only child were found to be nonsignificant factors for nursing students’ career maturity, aligning with previous research findings [44, 58].However, grade level did have a notable association with career maturity, with higher grades corresponding to increased levels of career maturity among nursing students, supporting earlier results [58, 59].In the context of Chinese nursing college education, a three-phase curriculum system is implemented, progressing from professional foundation courses to professional integrated skills courses and then to the clinical application of these skills. This structure allows students in higher grades to accumulate more profession-specific knowledge and clinical experience, thereby enhancing their understanding of nursing practice and supporting higher levels of career maturity. Some studies have reported a decline in career maturity during the final year due to job-seeking stress [52], which differs from our findings. This discrepancy may relate to differences in sample composition, timing of data collection, or variations in curriculum design and clinical exposure across institutions.Taken together, the observed developmental trend in this study is consistent with Super’s career development theory, which emphasizes stage-related growth in career readiness, and complements the SCCT framework by highlighting how educational stage interacts with cognitive and motivational mechanisms to shape career maturity.Therefore, it is crucial for nursing educators to provide students with comprehensive information and enhance their vocational skills and knowledge. By tailoring their approach to the specific needs of students at different grade levels, educators can effectively promote the development of career maturity in nursing students.

In summary, nursing college students exhibit a moderate level of career maturity. Work values, CDMSE, and locus of control were all associated with career maturity, with CDMSE showing the strongest association and chance showing a robust negative association. By simultaneously modeling these relations and accounting for measurement error, the SEM provides a more robust test than correlations or regression alone and highlights how psychological resources and control beliefs operate jointly rather than independently. These findings underscore the value of educational strategies that strengthen decision-making self-efficacy, support positive value appraisal of nursing work, and cultivate a greater sense of personal agency in career development.

### Limitations of this study

Using a large sample from various regions of China, this study explored associations among nursing college students’ career maturity, work values, CDMSE, and locus of control. However, this study has some limitations that should be addressed. First, the generalizability of the findings is limited because convenience sampling was used and participants were recruited from only five institutions. These sites were selected for feasibility of coordinated data collection and because they had established undergraduate nursing programs; however, the sample may not represent nursing students from other regions or institutional types in China. In addition, the sample was predominantly female (93.5%), which reflects the nursing workforce but may limit applicability to male nursing students. Future studies should recruit more diverse institutions and more gender-balanced samples where feasible.Second, the cross-sectional design of this study limits the ability to establish causal relationships between career maturity, work values, CDMSE, and locus of control. Longitudinal studies are recommended to better elucidate these causal dynamics.Third, the factors examined explained only part of the variance in career maturity, leaving a substantial proportion unexplained. Future research should investigate additional determinants, such as family expectations, professional identity, or institutional characteristics, and may include other health professional disciplines to examine whether these associations hold across educational contexts.

## Conclusion

This study demonstrates that career maturity among Chinese undergraduate nursing students is shaped by the joint influence of work values, career decision-making self-efficacy, and locus of control. Rather than acting independently, these factors appear to operate through interconnected psychological mechanisms, with decision-making self-efficacy playing a central role in translating values and control beliefs into mature career planning. By situating these findings within the Social Cognitive Career Theory framework, this study extends existing nursing education research by clarifying how cognitive and motivational resources jointly contribute to career maturity. These insights underscore the importance of addressing both internal psychological resources and contextual beliefs when supporting nursing students’ career development.

### Implications for healthcare and nursing

Informed by the structural equation modeling findings and guided by Social Cognitive Career Theory, the following recommendations translate the empirical results into actionable implications for nursing education and policy.First, at the curriculum level, nursing programs should integrate structured career development components across academic years. Early-stage courses may emphasize value clarification and professional socialization, whereas later-stage courses should focus on career planning, goal setting, and decision-making skills.Second, at the counseling and student support level, interventions should prioritize strengthening career decision-making self-efficacy through guided self-appraisal, improved access to occupational information, and support for realistic goal formulation. Empowerment-based approaches may be especially beneficial for students with strong external control orientations.Third, at the institutional and policy level, nursing schools and education policymakers should consider implementing systematic career maturity assessments, mentorship programs, and supportive learning environments to enhance students’ internal locus of control and long-term career stability.

## Data Availability

Please contact corresponding author for data requests.
